# Effect of the Pore Size on the Biological Activity of β-Ca_3_(PO_4_)_2_-Based Resorbable Macroporous Ceramic Materials Obtained by Photopolymerization

**DOI:** 10.1134/S0036023621110061

**Published:** 2021-11-18

**Authors:** P. V. Evdokimov, S. A. Tikhonova, A. K. Kiseleva, Ya. Yu. Filippov, E. S. Novoseletskaya, A. Yu. Efimenko, V. I. Putlayev

**Affiliations:** 1 Kurnakov Institute of General and Inorganic Chemistry, Russian Academy of Sciences, 119991 Moscow, Russia; 2 Moscow State University, 119991 Moscow, Russia

**Keywords:** macroporosity, bioceramics, calcium phosphates

## Abstract

The effect of the pore size of macroporous ceramic materials based on β-Ca_3_(PO_4_)_2_ on their biological activity was studied. The formation conditions of macroporous ceramics with a porosity of >50% and a specified pore size were determined. The effect of components of the light-curing emulsion on the pore size in the final macroporous ceramics was studied. The biocompatibility of β-Ca_3_(PO_4_)_2_-based macroporous ceramics was demonstrated in in vitro biomedical assays. The effect of pore size of macroporous ceramic materials on mesenchymal stromal cell proliferation and viability was established.

## INTRODUCTION

A highly relevant trend of modern medicinal materials science is development of personalized materials for bone defect regeneration [1] to be used for restoration of damaged tissues, their environment, and functions. Ideally, these implants must have a controlled resorption rate for the use in various clinical cases [2].

The idea to mimic the bone by a material that would be similar in the chemical composition [3], architectonics [4], and mechanical functions [5] is still considered relevant. Regenerative therapies use tissue-engineered constructs to attempt to restore the biological functions of particular tissues (bone tissue, skin, muscles, etc.). The design of such constructs for the treatment of bone defects is especially important if the defect size exceeds the critical size and the capability of the body is insufficient to restore the bone tissue [6].

Osteoconductive bioceramics with open porosity is a promising material for various fields of medicine in which osteoplasty is required [7]. Exhibiting osteoconductive properties requires that materials have open porosity of at least 40% [8]. The relatively high porosity markedly changes the mechanical properties of the bioceramic implants [9], particularly, decreases the strength and other mechanical characteristics needed for surgical manipulations during implantation. Numerous methods for the production of porous ceramic materials can be found in the literature: foaming of suspensions [10], direct [11] and inverse [12] replication, burn-up of inclusions [13], and so on. For efficient intergrowth of the newly formed bone tissue into this material, the material should have intersecting pores with a size of not less than 100–500 μm and channels connecting the pores with a diameter of not less than 50 μm [14–18].

Despite numerous studies demonstrating the effect of the overall porosity of materials on the rate and intergrowth volume of new bone tissue, there is still no common opinion about the optimal porosity. It was shown that the presence of significant porosity in the material has a beneficial effect on the osteogenesis, as it increases the overall permeability of the material and the contact area of the material with the newly formed bone tissue, which increases the adsorption properties for bone-inducing factors [19, 20]. Generally, two classes of pores required for bone-replacing materials are distinguished: micropores (<10 μm) and macropores (>100 μm) [21, 22]. It was shown that implants with >300 μm pores demonstrate high osteogenesis [15, 23]. Apart from the pore volume and size, osteogenesis depends on the curvature of the surface, which can also affect the activity of osteoclasts [24–27].

The purpose of this study is to elucidate the influence of the pore size on the biological activity of macroporous ceramic materials based on tricalcium phosphate and to develop a method for the production of macroporous ceramic materials with a controlled porosity. The method includes UV radiation-induced photopolymerization of oil-in-water emulsions containing tricalcium phosphate powder in the aqueous phase and subsequent heat treatment of the composite.

## EXPERIMENTAL

The light-curing emulsions were obtained using the following chemicals: tricalcium phosphate (TCP) Ca_3_(PO_4_)_2_, PEGDA-700 (polyethylene glycol diacrylate with a molecular weight of 700 Da, Sigma Aldrich, Germany), distilled water, paraffin oil (Sigma Aldrich, Germany), polyacrylic acid (Sigma Aldrich, Germany), polyethoxylated castor oil as the emulsifier (Sigma Aldrich, Germany), and diphenyl(2,4,6-trimethylbenzoyl)phosphine oxide as the photoinitiator (Sigma Aldrich, Germany).

TCP was obtained by solid-state synthesis from calcium carbonate CaCO_3_ (99.0%, Sigma Aldrich, Germany) and calcium pyrophosphate (CPP) Ca_2_P_2_O_7_, and CPP was prepared by thermal decomposition of monocalcium phosphate monohydrate (brushite) CaHPO_4_·2H_2_O.

Brushite CaHPO_4_·2H_2_O was precipitated according to reaction (1) by pouring together solutions with equimolar contents of Ca(NO_3_)_2_·4H_2_O (99.0%, Sigma Aldrich, Germany) and (NH_4_)_2_HPO_4_, (99.0%, Fluka Analytical, Germany) followed by stirring for 15 min.

1 $$\begin{gathered} {\text{Ca}}{{\left( {{\text{N}}{{{\text{O}}}_{{\text{3}}}}} \right)}_{{\text{2}}}}{\text{ + }}{{\left( {{\text{N}}{{{\text{H}}}_{{\text{4}}}}} \right)}_{{\text{2}}}}{\text{HP}}{{{\text{O}}}_{{\text{4}}}}{\text{ + 2}}{{{\text{H}}}_{{\text{2}}}}{\text{O}} \\ {\text{ = }}\,\,{\text{CaHP}}{{{\text{O}}}_{{\text{4}}}}{{\cdot}}{\text{2}}{{{\text{H}}}_{{\text{2}}}}{\text{O + 2N}}{{{\text{H}}}_{{\text{4}}}}{\text{N}}{{{\text{O}}}_{{\text{3}}}}{\text{,}} \\ \end{gathered} $$

The resulting precipitate was collected on a Büchner funnel, dried, and heat-treated at 500°C. During heat treatment, gradual dehydration of brushite took place according to Eq. (2):

2 $${\text{2CaHP}}{{{\text{O}}}_{{\text{4}}}} \cdot {\text{2}}{{{\text{H}}}_{{\text{2}}}}{\text{O}}\,\,\xrightarrow{{{{500^\circ \text{C}, 6}}\,{\text{h }}}}\,\,{\text{C}}{{{\text{a}}}_{{\text{2}}}}{{{\text{P}}}_{{\text{2}}}}{{{\text{O}}}_{{\text{7}}}} + {\text{3}}{{{\text{H}}}_{{\text{2}}}}{\text{O}}{\text{.}}$$

Calcium pyrophosphate and calcium carbonate CaCO_3_ were mixed in 1 : 1 ratio in a Pulverisette planetary mill (Fritsch, Germany) for 15 min; the milling bodies : powder : acetone ratio was 5 : 1 : 1 by weight. After milling, the mixtures were dried in air and then heat-treated at 900°C for 6 h. The following reaction took place during the heat treatment:

3 $${\text{C}}{{{\text{a}}}_{{\text{2}}}}{{{\text{P}}}_{{\text{2}}}}{{{\text{O}}}_{{\text{7}}}} + {\text{CaC}}{{{\text{O}}}_{{\text{3}}}} \to {\text{ C}}{{{\text{a}}}_{{\text{3}}}}{{\left( {{\text{P}}{{{\text{O}}}_{{\text{4}}}}} \right)}_{{\text{2}}}}{\text{ + C}}{{{\text{O}}}_{{\text{2}}}} \uparrow {\text{.}}$$

The emulsions were prepared as follows: the specified amount of the emulsifier was dissolved in distilled water and mixed with an equal amount of PEGDA solution containing the photoinitiator. Then a TCP powder (30 vol %) and paraffin oil (50 vol %) were added, and a mixture was mixed in a SpeedMixer DAC 150 laboratory planetary mixer (Germany).

The composites were obtained by filling the light-cured emulsion into a transparent silicone mold with a cylindrical hole and subsequent UV irradiation for 20 min. The macroporous ceramic samples were formed as cylinders (6 mm in diameter, 12 mm in height) by heat treatment of polymer–TCP powder–paraffin oil composites.

Powder X-ray diffraction studies were carried out on a Rigaku D/Max-2500 diffractometer with a rotating anode (Japan) in the reflection mode (Bragg–Brentano geometry) using Cu*K*_α_ radiation (average wavelength of 1.54183 Å).

The particle size distribution was determined by static light scattering using an Analysette 22 MicroTec plus laser diffraction analyzer (Fritsch, Germany). The results were derived from the frequency distribution of the Doppler shift of the laser beam by means of the MassControl software.

The microstructures of the composites and ceramics were examined on a Leo Supra 50VP field-emission scanning electron microscope (SEM) (Carl Zeiss, Germany).

Thermogravimetric analysis and differential thermal analysis of composites were carried out on a STA 409 PC Luxx simultaneous thermal analyzer (Netzsch, Germany) with vertical sample loading. The measurements were performed in air.

The in vitro assays were done using ASC52telo (hTERT-immortalized mesenchymal stem cells (MSC)) (ATCC® SCRC-4000^TM^). The MSC were cultured using the DMEM low glucose basal medium (Gibco, US) with addition of 10% fetal bovine serum (HyClone, US), 1% penicillin/streptomycin solution (HyClone, US), and 1% GlutaMAX-1 (Gibco, US). During culturing, conditions of 5% CO_2_ and 37°C were maintained. The medium was replaced every 3 days.

The test materials were sterilized in a dry heat sterilizer at 180°C for 60 min in SteriT® kraft paper bags (manufactured by Vinar research and production company, Russia). The quality of sterilization was checked using SteriTEST-Vl indicators (Vinar research and production company, Russia). Before seeding the cells, the samples were placed into the culture medium and incubated for 1 h. Then the medium was removed and the cells were seeded.

To estimate proliferation, MSC were seeded in a density of 40 thousand cells per well, incubated for 15 min at 37°C, and then the culture medium was added at the well edge. Then the cell viability was assessed on the 1st, 4th, and 7th days of culturing using the MTT assay. For this purpose, the tetrazolium dye 3-(4,5-dimethylthiazol-2-yl)-2,5-diphenyltetrazolium bromide (MTT) (Paneko, Russia) dissolved in sodium phosphate buffered saline (Paneko, Russia) to obtain a 5 mg/mL solution. The solution was passed through a filter with a pore size of 0.22 μm under sterile conditions. At each time point, the culture medium was replaced by a serum-free medium, and a sterile MTT solution was added in 4 : 1 ratio. The mixture was incubated for 2 hours under the above-described culturing conditions. After the incubation time was over, dimethyl sulfoxide was added into each well (AppliChem, USA) in 2 : 3 ratio to the mixture, and the final solution was transferred into a 96-well plate (250 μL per well). The absorbance was measured on a EnVision Multilabel Plate Reader (Perkin Elmer, USA) at λ = 595 and 630 nm.

The cell-containing samples were fixed in 10% neutral formalin and washed in a phosphate buffered saline (Paneko, Russia). Then dehydration was performed by successive treatment with ascending concentrations of ethanol. At the end, hexamethyldisilazane (EKOS-1, Russia) was added to the samples, and, after 30 min, the liquid was completely withdrawn, and the samples were dried under an exhaust hood at room temperature.

## RESULTS AND DISCUSSION

**Fig. 1.**
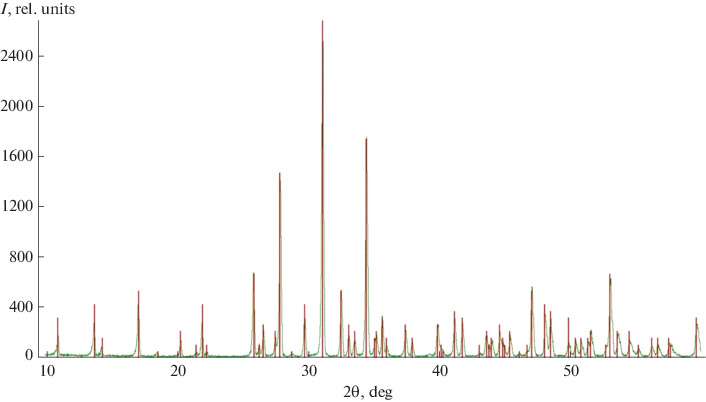
X-ray diffraction pattern of β-Ca_3_(PO_4_)_2_ synthesized at 900°C. The line diagram corresponds to the file # 70-2065 of ICDD PDF-2.

**Fig. 2.**
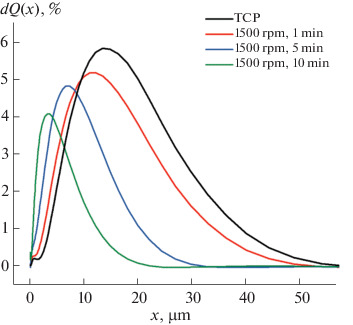
Differential particle size distribution curves for TCP particles after milling in a planetary mill for different time periods.

According to powder X-ray diffraction data (Fig. 1), the solid-state synthesis gave single phase TCP. The resulting powder was subjected to additional milling in acetone in the presence of a surfactant, which served as the filler to prepare light-cured emulsions, for surface modification. According to dynamic light scattering data, the use of surfactants stabilizes the particles and suppresses agglomeration at high milling speed and long milling time. The subsequent experiments were performed with a TCP powder with an average particle size of 3 μm (Table 1) and a fairly narrow size distribution (Fig. 2).

**Table 1.** Average pore size of TCP powder for different milling conditions

Speed, rpm	Average particle size, μm					
	milling without a surfactant			milling with a surfactant		
	1 min	5 min	10 min	1 min	5 min	10 min
500	12 ± 9	12 ± 7	12 ± 7	10 ± 11	7 ± 8	7 ± 4
1500	12 ± 6	7 ± 4	3 ± 2	8 ± 9	4 ± 2	3 ± 2
3000	8 ± 4	4 ± 4	6 ± 5	6 ± 4	3 ± 3	3 ± 2

**Fig. 3.**
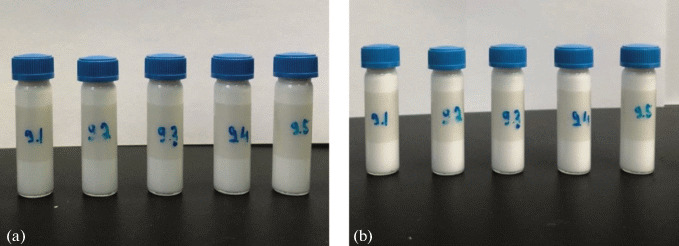
External appearance of suspensions with different emulsifier contents: (a) immediately after preparation, (b) a week later (the emulsifier content increases from 0 to 1 wt % from left to right).

Macroemulsions are complex, kinetically unstable systems. The presence of dispersed phase droplets with large diameters (10 to 1000 μm) results in phase separation with time. The light-curing “ceramic” macroemulsion has a complex composition: the dispersion medium is a suspension of TCP powder in water, which additionally complicates the formulation of a stable macroemulsion. As the content of emulsifier increases (from 0.05 to 1 wt %), the average size of oil droplets in the emulsions decreases from 500 to 14 μm, which finally leads to increasing viscosity of the emulsions, which remain stable and do not undergo phase separation within a week (Fig. 3).

**Fig. 4.**
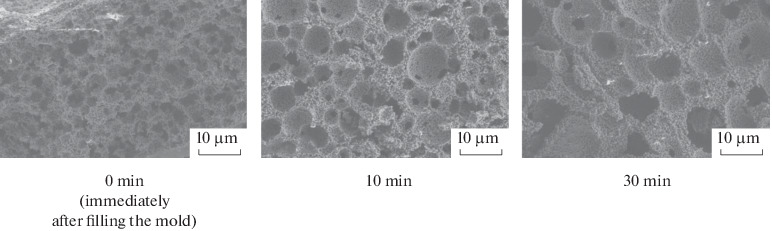
SEM data for TCP-based macroporous ceramic materials obtained by UV polymerization of an emulsion containing 0.25 wt % emulsifier after different time periods.

**Fig. 5.**
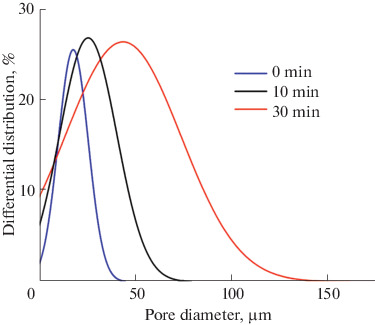
Pore size distribution for ceramic materials obtained from light-curing emulsions with different delays before UV polymerization.

The emulsions are prone to enlargement of droplets of the dispersed phase via primary coalescence and Ostwald ripening of large droplets, which absorb smaller droplets. In order to determine the emulsion stability with time and the possibility of retaining oil droplets of a specified size during storage, we studied the effect of time the emulsion is kept before UV irradiation on the pore size and size distribution of the final ceramic samples. For this purpose, an emulsion containing 0.25 wt % emulsifier was placed into a silicone mold to manufacture cylindrical composites and exposed to UV light after various time periods: 0 (immediately after the mold was filled), 10 min, and 30 min. Figure 4 shows the SEM data for ceramic materials obtained from these composites. One can clearly see an increase in the pore size accompanied by an increase in the thickness of the ceramic framework. Figure 5 shows the pore size distribution in ceramic materials. The average pore size increases and the pore size distribution is broadened, with the overall pore fraction being >55%.

**Fig. 6.**
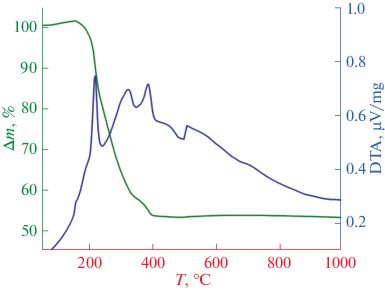
TG/DTA data for the oil–hydrogel/TCP composite obtained by photopolymerization.

Differential thermal analysis study of the removal of the organic component from the resulting oil–hydrogel/TCP composites (Fig. 6) showed that the use of emulsion produces a continuous network of the oil phase, which does not affect the hydrogel/TCP framework during heat treatment; this allows easy removal of paraffin oil from the composite. In turn, the highly porous composite framework has relatively thin walls (0 to 200 μm), which allows the use of more gentle temperature programs for polymer removal, despite numerous exothermic events during heating.

**Fig. 7.**
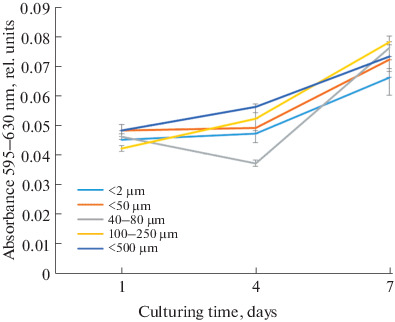
MSC proliferation on the surface samples with different pore sizes for 7 days. The data are given as medians (25.75%). The statistical significance for *p* < 0.05 is given according to the Kruskal–Wallis test using Dunn index for multiple comparison.

**Fig. 8.**
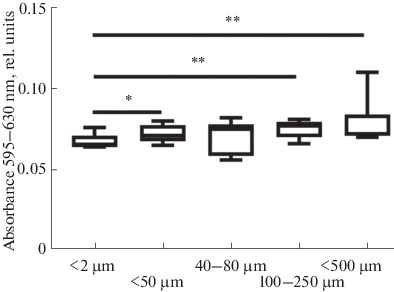
MSC proliferation on the surface samples with different pore sizes on the 7th day of the experiment. The data are given as medians (25.75%). The statistical significance between the groups for *(*p* < 0.05), **(*p*_value_ < 0.005) is given according to the Mann–Whitney test.

**Fig. 9.**
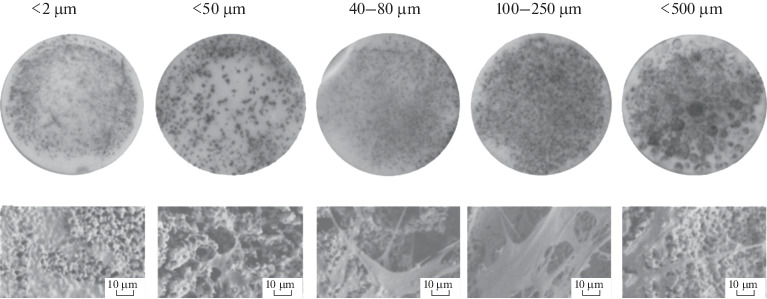
General view of “tablet” type samples on the 7th day of MSC culturing (upper panel photographs, contrasting with MTT assay) and cell morphology (lower panel, SEM images).

According to the results of the MTT assay evaluating cell proliferation and viability, all samples supported cell division throughout the experiment (Fig. 7). At the final point of the experiment, statistically significant differences were observed between samples with pore sizes of <50 μm, in the 100–250 μm range, and of <500 μm and the control sample with pore size <2 μm (Figs. 8, 9). According to SEM data, the cell morphology of ceramic samples with an average pore size of 100–250 μm differs from the control: they have more extensions, and, apparently, their synthetic function is more active, which is expected to significantly affect the cell differentiation in vivo. In the presence of larger pores (~500 μm) in the ceramic samples, MSC morphology is similar to that of the control, as cells spread over the whole pore surface.

## CONCLUSIONS

The presented conditions for the preparation of light-curing TCP-based dispersion systems give stable macroemulsions for manufacturing macroporous ceramics. By varying the emulsifier concentration, one can control the pore size in the final β-Ca_3_(PO_4_)_2_-based ceramics The variation of average pore size in ceramic materials based on tricalcium phosphate affects the proliferation and morphology of mesenchymal stromal cells. Macroporous ceramic materials with pores of 100–250 µm showed the most pronounced effect on the MSC proliferation and viability.
